# Graph neural network recommendation algorithm based on improved dual tower model

**DOI:** 10.1038/s41598-024-54376-3

**Published:** 2024-02-15

**Authors:** Qiang He, Xinkai Li, Biao Cai

**Affiliations:** 1 School of Mechanical and Electrical Engineering, Chengdu University of Technology, Chengdu, 610059 China; 2 School of Computer Science and Cyber Security, Chengdu University of Technology, Chengdu, 610059 China; 3 College of Industrial Technology, Chengdu University of Technology, Yibin, 644000 China

**Keywords:** Recommendation, Dual tower model, Graph neural network, Collaborative filtering, Mathematics and computing, Physics

## Abstract

In this era of information explosion, recommendation systems play a key role in helping users to uncover content of interest among massive amounts of information. Pursuing a breadth of recall while maintaining accuracy is a core challenge for current recommendation systems. In this paper, we propose a new recommendation algorithm model, the interactive higher-order dual tower (IHDT), which improves current models by adding interactivity and higher-order feature learning between the dual tower neural networks. A heterogeneous graph is constructed containing different types of nodes, such as users, items, and attributes, extracting richer feature representations through meta-paths. To achieve feature interaction, an interactive learning mechanism is introduced to inject relevant features between the user and project towers. Additionally, this method utilizes graph convolutional networks for higher-order feature learning, pooling the node embeddings of the twin towers to obtain enhanced end-user and item representations. IHDT was evaluated on the MovieLens dataset and outperformed multiple baseline methods. Ablation experiments verified the contribution of interactive learning and high-order GCN components.

## Introduction

Recommendation systems^1,2^ have an important role in helping users uncover interesting content from massive amounts of information in the context of the current information explosion era. Early methods were based on collaborative filtering^3^ and mainly relied on matrix decomposition^4–8^, such as hybrid algorithms based on Probs and Heats calculation modes^6^. However, collaborative filtering is based on users’ historical behavioral information and cannot effectively model auxiliary information such as social relationships and product attributes. Subsequently, researchers incorporated content features to overcome the cold start problem^9–11^.

Most early research efforts focused on homogeneous networks composed of nodes and edges of the same type. For example, Perozzi et al.^12^ proposed the deep walk model that combines random walk with the skip-gram model^13^. Subsequently, Grover et al.^14^ proposed depth-first and breadth-first wandering strategies to capture different network structure information by improving the wandering strategy of deep walk; both strategies are used in the Node2Vec model. The LINE model proposed by Tang et al.^15^ defines first-order and second-order similarities to learn the node representation of large-scale sparse networks. However, these three models are shallow, and the network representation they generate is not optimal because the captured network nodes are too close and full of local information. Graph neural network (GNN) models^16–39^ have recently brought new opportunities to recommendation systems, which can explicitly model high-order user–product interactions to enrich expressions^16,17^. For example, Berg et al.^18^ designed a recommendation method based on a graph autoencoder through message passing and aggregation. Wang et al.^19^ proposed that a spatial GNN would be superior to traditional collaborative filtering, such as NCF^20^. Sun et al.^21^ believed that a simple aggregation mechanism could not effectively utilize neighbor information, so they designed neighbor interactive aggregation.

The core challenge that current recommendation systems face is pursuing recall breadth without sacrificing accuracy. Due to its high efficiency in large-scale candidate record screening, the two-tower neural network model^40–44^ has received widespread attention. However, its user and product tower are trained independently and cannot effectively model the interaction between features, which results in poor recommendation accuracy. Recently, GNN models have been successfully used in recommendation systems, achieving significant performance improvements through high-order feature interaction.

We propose an interactive high-order twin-tower model (IHDT) that considers the speed advantage of the twin-tower model and the accuracy advantage of GNNs. This model is built on a heterogeneous graph of user products and uses an interactive learning mechanism to inject product (user) features related to users (products) into the corresponding encoder. The model uses high-order feature expression based on graph convolutional networks to aggregate multipored neighbor information of nodes and enhance the vector representation of users and products. Finally, the inner product between the augmented representations is calculated as the predicted value of the recommendation system. The effectiveness of the model was verified on public datasets, and the results show that IHDT achieves state-of-the-art performance comparable to multiple powerful benchmarks. The IHDT model thus offers interactive modeling of user products and graph-based high-order feature learning, and as such provides new ideas for large-scale recommendation systems considering both accuracy and diversity.

## Model

### Problem definition

Real-world data often contain multiple types of objects and their interactions, which makes it difficult to model the data using homogeneous information networks, as representing learning using a homogeneous information network captures only some features. However, these heterogeneous data can be naturally modeled using heterogeneous information networks, in which multiple types of objects and interactions can coexist, containing rich network structure information and semantic information. The relevant definitions are shown below.

#### Definition 1

*Information Network*. An information network is represented as ${\text{G}}=({\text{V}},{\text{E}},\mathrm{\varphi },\uppsi )$, consisting of a set of objects V, a set of links E, a mapping function of object types $\mathrm{\varphi }:{\text{V}}\to {\text{A}}$, and a mapping function of relationship types $\uppsi :{\text{E}}\to {\text{R}}$. A is the set of object types, and R is the set of relationship types.

#### Definition 2

*Homogeneous/Heterogeneous Information Network*. An information network is heterogeneous if the number of object types is $|{\text{A}}|>1$ or the number of relationship types is $|{\text{R}}|>1$. Otherwise, it is called a homogeneous information network.

**Figure 1:**
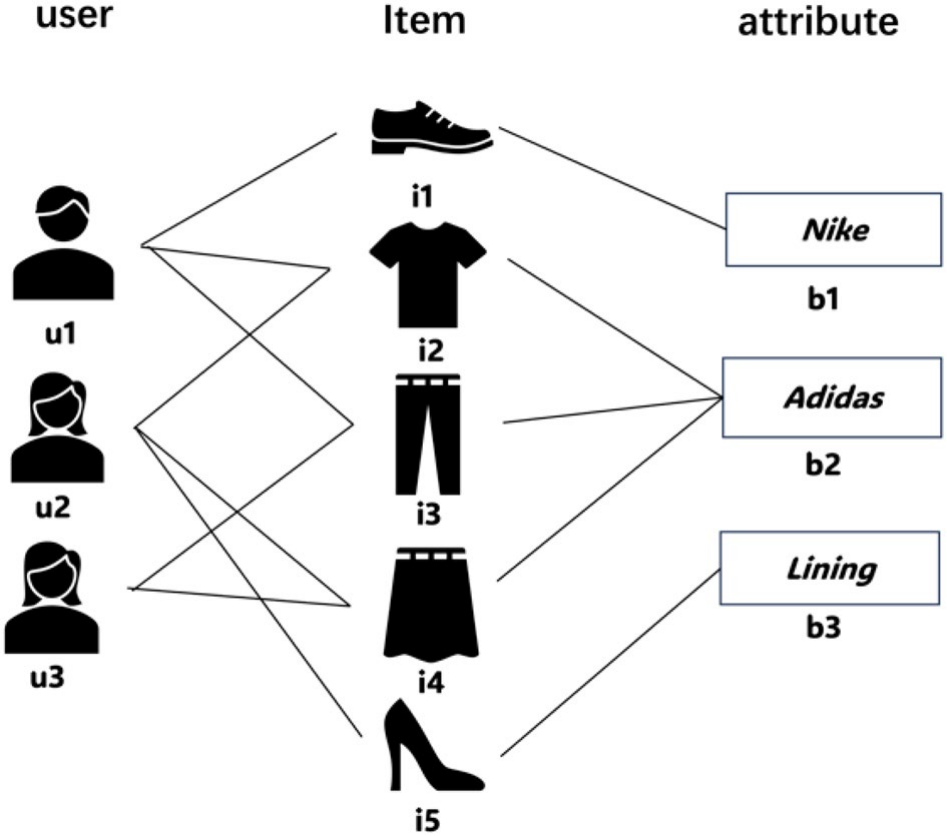
Heterogeneous information network.

A simple heterogeneous information network is shown in Fig. 1. It contains three types of nodes: user, item, and brand, and the relationships between them.

#### Definition 3

*Network Model*. The network model^45^ can be represented as ${{\text{T}}}_{{\text{G}}}=({\text{A}},{\text{R}})$. It is a directed graph with object type A as nodes and relationship type R as edges.

#### Definition 4

*Meta-path*. A meta-path is a path of two classes of objects, A and R, in the network pattern ${{\text{T}}}_{{\text{G}}}=({\text{A}},{\text{R}})$, which can be expressed as ${{\text{A}}}_{1}{\to }^{{{\text{R}}}_{1}}{{\text{A}}}_{2}{\to }^{{{\text{R}}}_{2}}...{{\to }^{{{\text{R}}}_{{\text{l}}}}{\text{A}}}_{{\text{l}}+1}$, representing a composite relationship.

#### Definition 5

*Recommendation Base Graph*. Traversing nodes and edges in the i-th meta-path pattern forms a subgraph, ${G}_{i}$, and finally merging all the obtained subgraphs to form the recommendation base graph G, i.e., ${\text{G}}=\bigcup {G}_{i}$.

**Figure 2:**
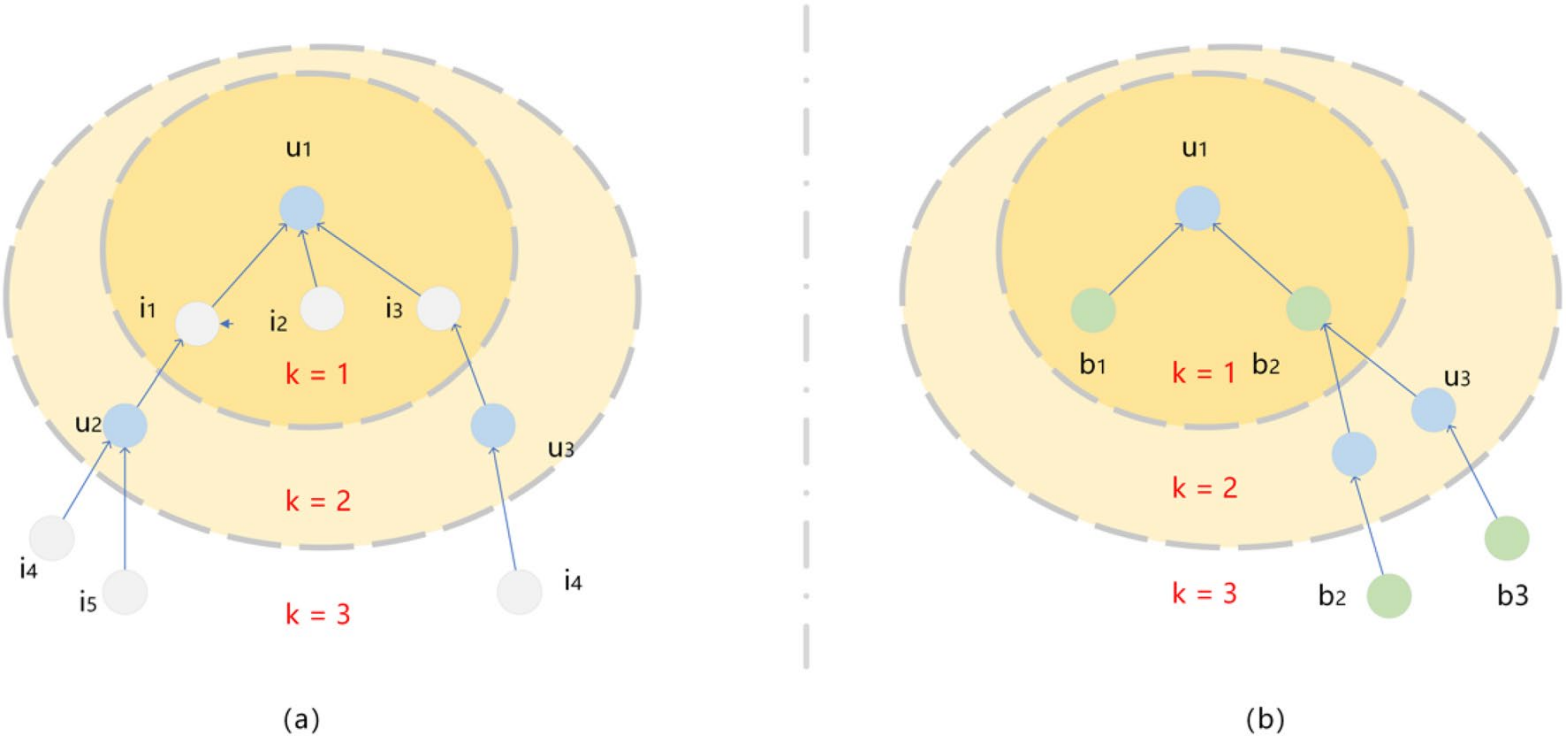
Two different higher-order connectivity of ${u}_{1}$. (**a**) Higher-order connectivity based on user–item meta-paths of ${u}_{1}$; (**b**) Higher-order connectivity based on user–attribute–item meta-paths of ${u}_{1}$.

In the heterogeneous information network, as shown in Fig. 2, different meta-paths are selected for user u_1_ to obtain different higher-order connectivity.

### IHDT model

**Figure 3:**
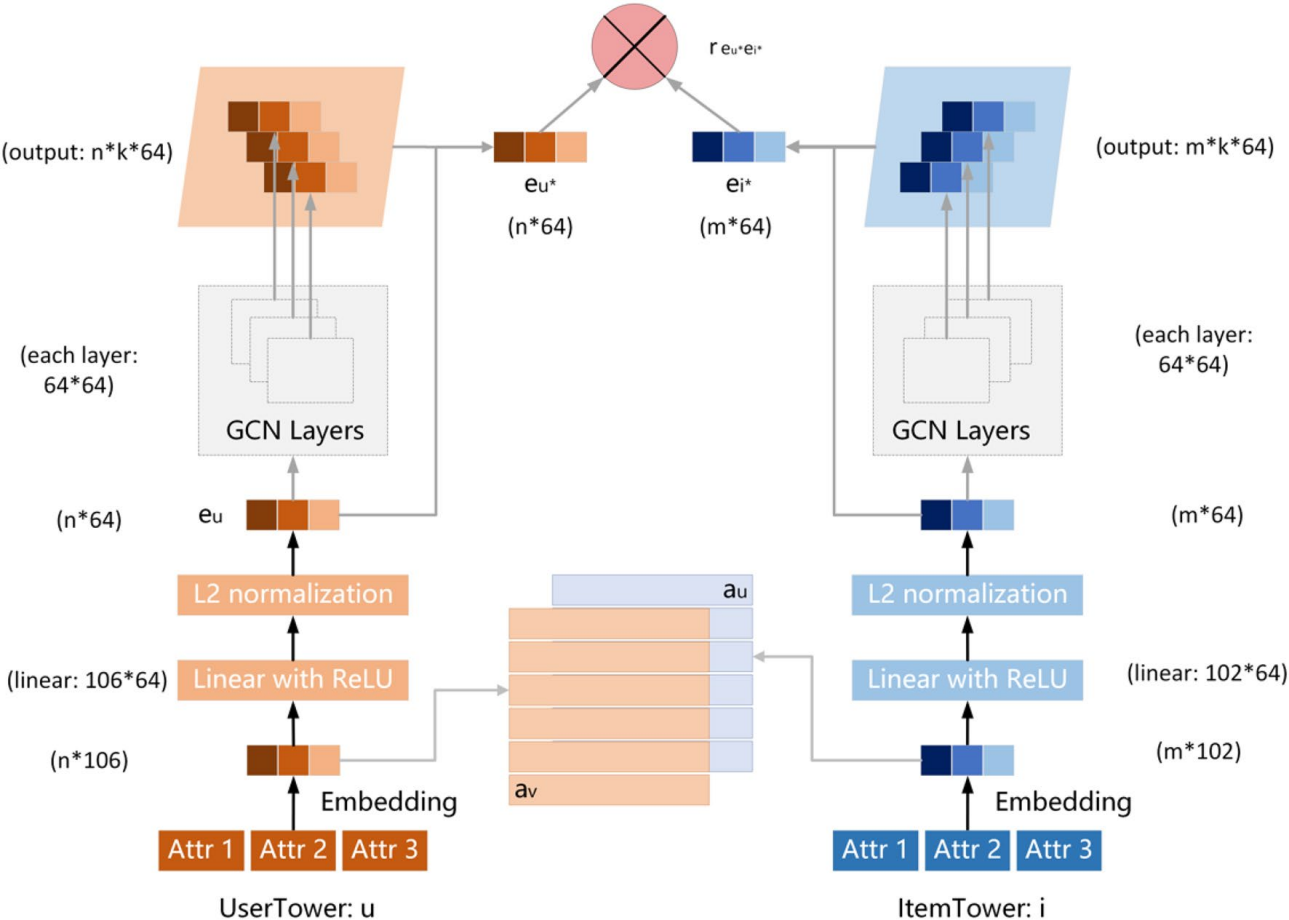
Interactive higher-order dual tower recommendation model (IHDT).

The IHDT model (see Fig. 3 for the architecture diagram) is based on interactive, high-order learning mechanisms to improve the dual tower model and the accuracy of the recommendation system. The IHDT model introduced in this paper learns node representations and applies them to recommendations in the following steps.

1. Select different-pattern meta-paths ${\Phi }_{i}$ in the data source to form different-pattern subgraphs ${G}_{i}$ and finally merge all subgraphs to form the recommendation base graph $G$ (i.e., ${\text{G}}=\bigcup {G}_{i}$).
2. Use random initialization or a pre-training model to obtain different node initialization expressions of $G$; the i-th user node is expressed as ${e}_{u}$, the k-th attribute of this user is expressed as ${e}_{u}^{k}$, the j-th item is expressed as ${e}_{i}$, and the h-th attribute of this item is expressed as ${e}_{i}^{h}$.
3. Using the interaction learning mechanism, the interaction mechanism expressions ${a}_{u}$ and ${a}_{v}$ of the user and item nodes are derived as the two inputs of the dual tower model.
4. Higher-order mechanism expression learning based on GCN converges and aggregates the multifaceted representations of nodes to obtain the final representations ${e}_{u}^{*}$ and ${e}_{i}^{*}$ of users and items.
5. Finally, the inner product of ${e}_{u}^{*}$ and ${e}_{i}^{*}$ is calculated and used as the final prediction value of the model.

## Method

The construction of the IHDT model, including implementing the interactive and high-order learning mechanisms, consists of the following steps.

### Interactive learning

The framework of the proposed model is shown in Fig. 3. Add user feature vector ${a}_{u}$ and product feature vector $a_{v}$ to the user and product input terminals, respectively. The user and item embedding representations $z_{u}^{i}$,$z_{v}^{j}$ $\in {\mathbb{R}}^{1 \times d^{\prime}}$ under specific user–item interaction are learned through the interaction mechanism G. The connection layer passes ${z}_{u}\in {\mathbb{R}}^{{\text{nu}}\times d}$ and ${z}_{v}$ $\in {\mathbb{R}}^{{\text{nv}}\times d}$ into the user tower and product tower, and outputs the vector representations of users and products $e_{u}$ and ${\text{e}}_{i}$:

1 $$\begin{array}{*{20}c} {{\text{z}}_{{\text{u}}}^{{\text{i}}} , {\text{z}}_{{\text{v}}}^{{\text{j}}} = G\left( {{\text{e}}_{{\text{i}}} \cdot {\text{a}}_{{\text{v }}} ,{\text{e}}_{{\text{j}}} \cdot {\text{a}}_{{\text{u }}} } \right),} \\ \end{array}$$

where $a_{u}$ and $a_{v}$ are derived from the information captured by the interaction behaviors in the corpus.

### High-order learning

The final output vectors ${e}_{u}$ and ${e}_{i}$ obtained in the dual tower model can only represent the first-order information of the current user (i.e., the rich feature information of the user (item)); the first-order interaction between the user and the item can be obtained in the dual tower model, but the convergence of higher-order information between the user and the item cannot be achieved. Using ${e}_{u}$ and ${e}_{i}$ as the input of GCN, first-order and high-order propagation aggregation rules are designed to obtain the final embedding representations of users and products ${{\text{e}}}_{{\text{u}}}^{*}$ and ${{\text{e}}}_{{\text{i}}}^{*}$. GCNs can achieve this purpose by propagating the convergence, ensuring the richness of ${e}_{u}$ and ${e}_{i}$ as well as the accuracy brought by the prediction:

2 $$\begin{array}{*{20}c} {e_{u}^{\left( 1 \right)} = {\text{LeakyReLU}}\left( {1/\sqrt {\left( {\left| {N_{u} } \right|\left| {N_{i} } \right|} \right)} \left( {W_{1} e_{i} + W_{2} \left( {e_{i} \odot e_{u} } \right)} \right)} \right),} \\ \end{array}$$

where ${\text{W}}_{1} ,{\text{W}}_{2} \in {\mathbb{R}}^{{d^{\prime} \times d}}$ is the trainable weight matrix used to extract useful propagation information, and $d^{\prime}$ is the transformation dimension, while $p_{ui}$ is set as the Laplacian matrix of the graph, and ${\mathcal{N}}_{u}$ and ${\mathcal{N}}_{i}$ denote the first-order neighbors of user *u* and item *i*, respectively.

On top of first-order propagation and convergence, higher-order propagation and convergence are embedded into the propagation layers by stacking l. Users (and items) can receive messages propagated from their L-Hop neighbors. Then, the final embedding representation of the final l-th layer can be obtained:

3 $$\begin{array}{c}{E}^{\left(l\right)}=LeakyReLU\left(\left(L+I\right){E}^{\left(l-1\right)}{W}_{1}^{\left(l-1\right)}\odot {E}^{\left(l-1\right)}{W}_{2}^{\left(l-1\right)}\right),\end{array}$$

where ${\text{E}}^{\left( l \right)} \in {\mathbb{R}}^{{\left( {N + M} \right)d_{l} }}$ is the representation obtained after the users, and items are embedded in the propagation convergence *l* layer. ${\text{E}}^{\left( 0 \right)}$ is ${\text{E}} = \left[ {\underbrace {{{\text{e}}_{{{\text{u}}_{1} }} , \ldots ,{\text{e}}_{{{\text{u}}_{{\text{N}}} }} }}_{{\text{users embeddings}}},\;\underbrace {{{\text{e}}_{{{\text{i}}_{1} }} , \ldots ,{\text{e}}_{{{\text{i}}_{{\text{M}}} }} }}_{{\text{item embeddings}}}} \right]$; I is the unit matrix. $\mathcal{L}$ represents the Laplacian matrix of the user–item bipartite graph.

### Model prediction

After propagation and convergence through *l* layers, the embedding representation of each user (item) is obtained at each layer, and the final user ${\mathbf{e}}_{u}^{*}$ and item ${\mathbf{e}}_{i}^{*}$ embedding representations are obtained by a simple join operation:

4 $$\begin{array}{c}{{\text{e}}}_{{\text{u}}}^{*}={{\text{e}}}_{{\text{u}}}^{\left(0\right)}\parallel \cdots \parallel {{\text{e}}}_{{\text{u}}}^{\left({\text{L}}\right)},{{\text{e}}}_{{\text{i}}}^{*}={{\text{e}}}_{{\text{i}}}^{\left(0\right)}\parallel \cdots \parallel {{\text{e}}}_{{\text{i}}}^{\left({\text{L}}\right)}.\end{array}$$

Then, preference prediction is performed by computing the inner product of the final embedding representations of users and items:

5 $$\begin{array}{c}{\widehat{{\text{y}}}}_{ }={{\text{e}}}_{{\text{u}}}^{*\mathrm{\top }}{{\text{e}}}_{{\text{i}}}^{*}.\end{array}$$

Finally, model optimization is performed based on the Bayesian personalized ranking (BPR) loss:

6 $$\begin{array}{*{20}c} {{\text{Loss}} = \sum\nolimits_{{\left( {{\text{u}},{\text{I}},{\text{i}}^{ - } } \right) \in {\text{O}}}} { - {\text{ln}}\left( {{\hat{\text{r}}}_{{{\text{u}},{\text{i}}}} - {\hat{\text{r}}}_{{{\text{u}},{\text{i}}^{ - } }} } \right),} } \\ \end{array}$$

where *O* represents sample set ${\text{u}},{\text{ I}}$ represents positive samples, and $u,i^{ - }$ represents negative samples. Finally, the above BPR loss function can be minimized, the parameters in the whole model can be optimized end-to-end in the form of backpropagation, and all parameters converge to a fixed value with the optimization process of the model.

### Overall process

According to the above, the overall training process of the IHDT model is as follows.

Table 4-1 Training process of IHDT.

**Algorithm 4.1:**
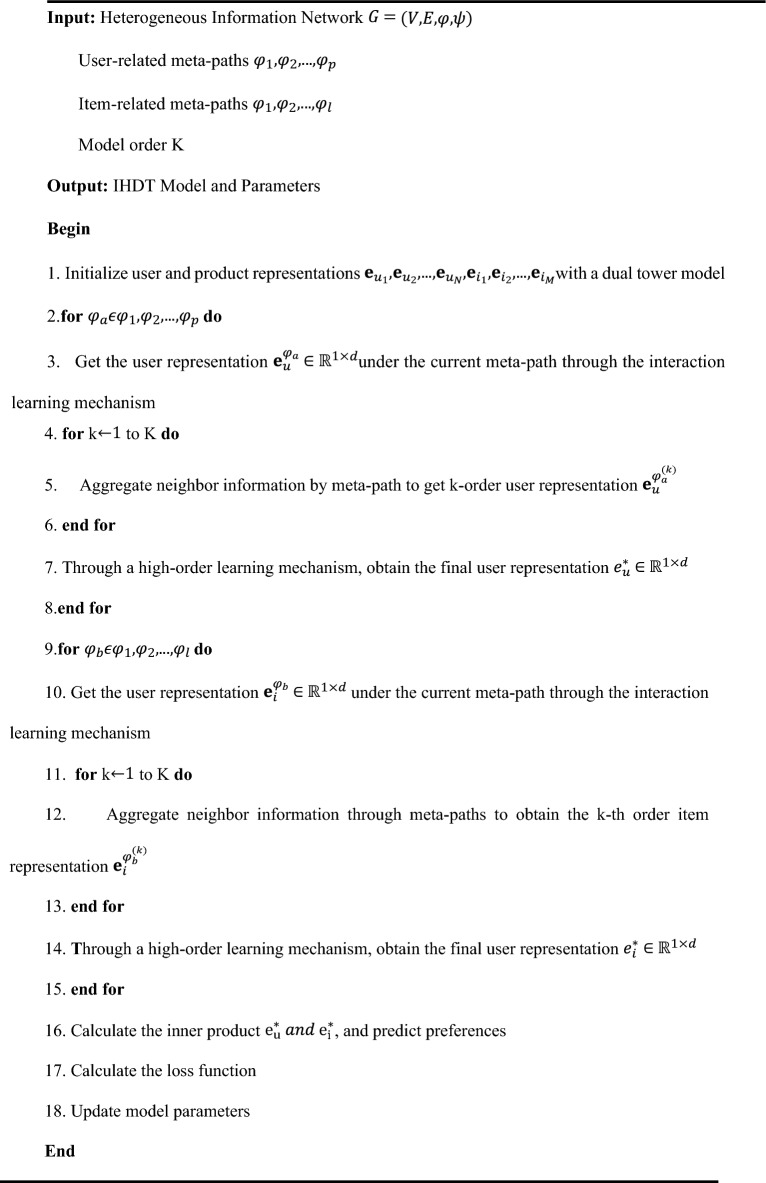
Training process of IHDT

## Experimental setup

### Dataset description

Experiments were conducted on two real-world datasets to evaluate the proposed IHDT model. The effectiveness of the IHDT model was evaluated by experiments conducted on two benchmark datasets, MovieLens-1M and MovieLens-10M. The MovieLens dataset was provided by the University of Minnesota’s Group Lens Project. The datasets are rated on a five-point scale, from 1 to 5, representing the user’s interest in the movie. They are all publicly desensitized and accessible and vary in domain, size, and sparsity. The statistical information of the datasets is shown in Table 1.

**Table 1:** Dataset description.

Dataset	Users	Item	Interaction	Sparsity
MovieLens-1M	$6040$	$3883$	$988129$	$4.213*{10}^{-2}$
MovieLens-10M	$69878$	$10677$	$10000054$	$1.34*{10}^{-2}$

For each dataset, 90% of the historical interactions of each user were randomly selected to form the training set, and the rest were used as the test set. Each pair of training and test sets is complementary, and recombining them can yield the initial dataset. From the training set, 10% of the interactions were randomly selected as the validation set to tune the hyperparameters. Each observed user–item interaction was treated as a positive instance. Then, a negative sampling strategy was used to pair it with a negative item the user had not used before.

### Evaluation metrics

For each user in the test set, we considered all items with which the user had no interaction as negative samples and items that the user had interactions with as positive samples. We utilized four commonly used performance evaluation metrics: precision, recall, normalized discounted cumulative gain (NDCG), and hit rate (HR).

We briefly introduce these metrics as follows.

Precision@K is used to evaluate the proportion of products related to the user among the top K products recommended to the user. It is calculated as follows:

7 $$\begin{array}{c}Precision\left({\text{K}}\right)=\frac{1}{{\text{m}}}{\sum }_{{\text{i}}=1}^{{\text{m}}}\frac{{{\text{d}}}_{{\text{i}}}\left({\text{L}}\right)}{{\text{L}}},\end{array}$$

where ${d}_{i}(K)$ is the intersection of the top $K$ products recommended to the user and the products in the test set that the user interacts with.

Recall@K is used to evaluate the proportion of the products that are related to the user to those that are recommended to the user:

8 $$\begin{array}{c}Recall\left({\text{K}}\right)=\frac{1}{{\text{m}}}{\sum }_{{\text{i}}=1}^{{\text{m}}}\frac{{{\text{d}}}_{{\text{i}}}\left({\text{L}}\right)}{{\text{D}}\left({\text{i}}\right)}.\end{array}$$

NDCG@K is used to evaluate the accuracy of the ranking results. Assuming that the length of the recommendation list is $K$, NDCG@K shows the gap between the ranking list and the real user interaction list. It is calculated as follows:

9 $$\begin{array}{c}NDCG@K={{\text{Z}}}_{{\text{k}}}\sum_{{\text{k}}=1}^{{\text{K}}} \frac{{2}^{{{\text{r}}}_{{\text{k}}-1}}}{{{\text{log}}}_{2}\left({\text{k}}+1\right)},\end{array}$$

where ${r}_{k}=1$ indicates that the *K*th item is the item that the user favors; otherwise, ${r}_{k}=0$. ${Z}_{k}$ is a normalization constant.

HR@K is a commonly used indicator to measure recall rate and is calculated as follows:

10 $$\begin{array}{c}HR@K=\frac{1}{{\text{N}}}\sum_{{\text{i}}=1}^{{\text{N}}} {\text{hits}}\left({\text{i}}\right),\end{array}$$

where $N$ is the total number of users, and $hits(i)$ represents whether the value accessed by the *i-*th user is in the recommended list. If yes, then $hits(i)$ equals 1; otherwise, it is 0.

### Baselines

The proposed IHDT was compared with the following top-k recommendation algorithms to demonstrate its effectiveness.

*MF*^4^. This is a matrix decomposition optimized by Bayesian personalized ranking (BPR) loss, which uses only the direct user–item interaction as the objective value of the interaction function.

*DMF*^46^. This method is a matrix decomposition model with a neural network architecture. A user–item matrix with explicit ratings and non-preference implicit feedback is constructed and used as input. A deep structure learning architecture is proposed to learn a generic low-dimensional space representing users and items.

*GCMC*^18^. This approach considers the matrix decomposition of recommendation systems from a link prediction perspective, represented by a bipartite user–item graph with labeled edges indicating the observed ratings. This way, a graph autoencoder framework is proposed based on micro-message transferable on bi-directional interaction graphs.

*NeuMF*^47^. This approach is an advanced neural CF model that uses multiple hidden layers above the element level and connections of user and item embeddings to capture their nonlinear feature interactions.

*ConvNCF*^48^. This method uses outer products to explicitly model pairwise correlations between embedding space dimensions. A more expressive and semantically sound 2-D interaction graph is obtained using the outer product on top of the embedding layer. On top of the interaction graph, a convolutional neural network (CNN) is used to learn higher-order correlations between the embedding dimensions.

*NGCF*^49^. This approach can explicitly encode the higher-order user–item interactions into the representation vector, effectively injecting collaborative signals into the embedding process in an explicit way to improve the representation and, thus, the overall recommendation.

*LightGCN*^50^ This method removes the feature transformation and nonlinear activation in GCN, making the model more concise and efficient. LightGCN only retains the neighbor aggregation operation of GCN, that is, layer-by-layer propagation and aggregation through the user–item interaction matrix. At the same time, a new vertical regularization term is introduced to punish overly complex user and item representations to prevent overfitting. In terms of efficiency, a sparse graph is constructed by discarding some connections to speed up training and inference. Additionally, two dropping strategies, random dropping and weighted sampling, are introduced. LightGCN generally achieves the best balance of accuracy and efficiency by designing a simple model and regularization strategy. It retains the key mechanisms GCN requires while removing unnecessary components, which is its main advantage over other GCN models.

BM3*^51^ This method is different from the previous method in that it is a multi-modal recommendation method. It designs a multi-modal contrastive loss function that simultaneously optimizes three goals: reconstructing user-item interaction graphs, aligning learned features between different modalities, and reducing the gap between different augmented view representations from a specific modality. dissimilarity. (*Note: The dataset we tested is a traditional recommendation system dataset which does not provide much additional information for multi-modal learning, so the effect may not ideal. This test is only to illustrate the multi-modal recommendation model does not have advantages for traditional recommendation tasks).

### Parameter setting

This study implemented the IHDT model in TensorFlow and Pytorch. The parameters were randomly initialized in TensorFlow for the improved dual tower model, and the number of connected layers was set to two by default. The output vector in the dual tower model was used as the initialization parameter of IHDT in Pytorch. The embedding dimensions of the model default were set to 64, the normalization coefficient was set to 1e−5 by default, the learning rate was uniformly set to 1e−4, the node loss ratio was set to 0.1, the message loss ratio was set to 0.1, the number of layers was set to 3, the layers in the Movielens-1M dataset batch size were set to 1024, the epoch was set to 1000, the dataset batch size in the MovieLens-10M was set to 4096, and epoch was set to 400. Additionally, an early stop policy was set (i.e., if the evaluation indicator recall did not increase within 50 consecutive epochs, it was stopped early).

## Experimental results and analysis

### Comparison of experimental results

Table 2 shows the results of Top-20 recommendations on MovieLens-1M and MovieLens-10M datasets for the IHDT and other benchmark algorithms described in this paper. The table shows that the IHDT algorithm outperformed the comparison algorithms in overall performance and was superior to the comparison algorithms for all indicators. The accuracy, recall, and NDCG were all improved.

**Table 2:** Algorithm @ 20 Performance of models with Top-20 on MovieLens dataset.

Model	Movielens-1M				Movielens-10M			
	Pre@20	Recall@20	NDCG@20	HR@20	Pre@20	Recall@20	NDCG@20	HR@20
MF (2009)	0.1636	0.2493	0.2661	0.8369	0.1458	0.3147	0.2862	0.8158
DMF (2017)	0.1566	0.2486	0.2540	0.8384	0.1286	0.2778	0.2495	0.7752
GCMC (2017)	0.1629	0.2548	0.2647	0.8429	–	–	–	
NeuMF (2021)	0.1553	0.2369	0.2465	0.8248	0.1458	0.3104	0.2862	0.8068
ConvNCF (2019)	0.0826	0.1134	0.1229	0.6240	0.1288	0.2642	0.2421	0.7802
NGCF (2019)	0.1643	0.2640	0.2711	0.8503	0.1736	0.3500	0.3345	0.8797
LightGCN (2020)	0.1831	**0.2789**	**0.3009**		0.1722	0.3457	0.3333	
BM3(2023)	0.1000	0.1516	0.1580		0.0983	0.2310	0.1949	
IHDT	**0.1852**	0.27210	0.2901	**0.8553**	**0.1742**	**0.3502**	**0.3350**	**0.8812**

Significant values are in bold.

Table 2 shows that the IHDT algorithm considerably improved over the benchmark algorithms. The IHDT and NGCF algorithms also performed better. This paper speculates that it may be due to the high sparsity of the MovieLens-10M dataset used in the study. Both the IHDT and NGCF models could aggregate the feature information of high-order neighbors. IHDT in the dual tower model also performed deep semantic interaction, so it still performed well with sparser data. MF and DMF were less effective on sparse datasets. Meanwhile, although NeuMF could also aggregate neighbor information, the converged information was insufficient for higher-order neighbors, resulting in moderate performance.

### Analysis of recommendation list length impact

After completing the basic experiments to investigate the effect of different recommendation list lengths on model performance, we continued to compare the model’s performance with different recommendation list lengths on the dataset of the basic experiment. The results of this experiment, which increased the recommendation list length from 10 to 100 while keeping other variables constant, are as follows.

**Figure 4:**
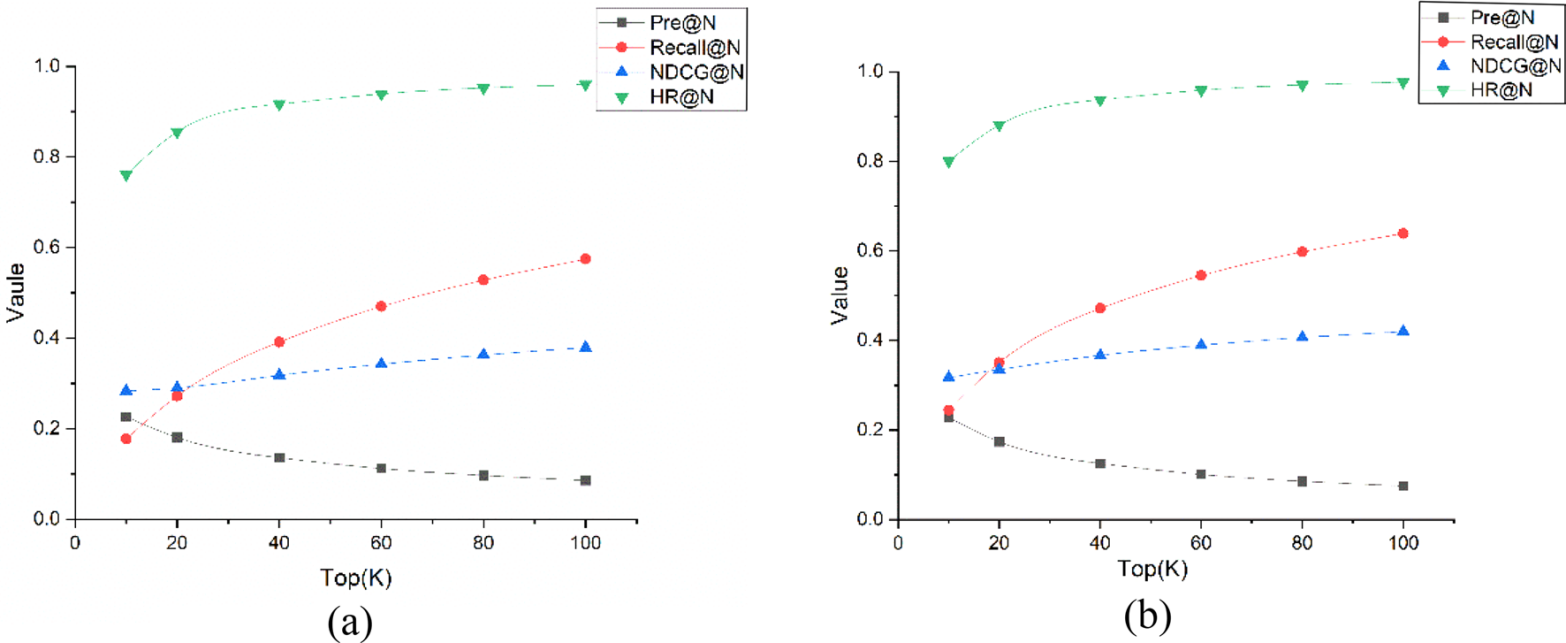
Performance under different datasets and recommended list lengths.

Figure 4 shows the performance trends of the IHDT model on Movielens-1M and MovielensLens-10M datasets for recommendation list lengths from 10 to 100 using line graphs. Based on the above experimental results, it was found that as the recommendation list length increased, both the recall indicator and NDCG indicator showed an increasing trend; the recall indicator increased significantly. The precision indicator decreased smoothly, indicating that the algorithm performed well when the recommendation list length was increased.

### Parameter sensitivity study

When the IHDT model was applied to a recommendation system, the main parameters included the number of model layers, the dimensionality d of the user and item representation vector (embedding), the learning rate (lr), and the L2 regularization coefficient. Taking the Movielens-1M dataset as an example, this study investigated the parameter variation of the IHDT algorithm with different parameter values.

#### Number of model layers

The method in this paper selects the 1st to Kth-order higher-order neighbor information for fusion. To verify the influence of the selected order on the experimental results, different orders were selected for the Movielen-1M dataset. The experimental results are shown in Table 3.

**Table 3:** Performance of models with different orders on Movielens-1M dataset.

Model Kth	Pre@20	Recall@20N	NDCG@20	HR@20
IHDT-1	0.16571	0.26601	0.27298	0.85262
IHDT-2	0.16482	0.26472	0.27139	0.85162
IHDT-3	**0.18052**	**0.27210**	**0.29007**	**0.85530**
IHDT-4	0.12378	0.25893	0.26543	0.84702

Significant values are in bold.

The details in Table 3 reflect that when the model order is small, increasing the order of the model improves the model performance effectively, and the performance of IHDT-3 is much higher than that of IHDT-1 and IHDT-2. The high-order connection better captured the collaborative relationship between node messages, improved the node feature representation, and improved the model performance; when the model order continued to increase, there was a dramatic decrease in the model performance. The table shows that the model performance is lowest when the IHDT model order is 4. This may be due to overfitting caused by introducing noisy information into the representation learning that could be caused by applying an excessively deep architecture. Therefore, it is necessary to select the appropriate model order to determine the maximum performance of the model.

#### Dimensionality of user and item representation vectors

For the dimension selection of the final representation vector of users and items, the dimensions of the final representation vector were 32, 64, and 128. Table 4 shows the experimental results.

**Table 4:** Algorithm performance on Movielens-1M dataset under different representation vector dimensions.

Dimensionality	32	64	128
Pre@20	0.15840	**0.18052**	0.16081
Recall@20	0.25278	**0.27210**	0.26131
NDCG@20	0.25912	**0.29007**	0.26478
HR@20	0.84089	**0.85530**	0.85265

Significant values are in bold.

Based on the experimental data in Table 4, the following findings are made: the IHDT indicators do not trend gradually upward with increasing dimensionality of the representation vector. The best performance of the proposed model was when the value of dimensionality was 64, indicating that the model needs sufficient dimensionality to encode the preferences of users or items. The model’s predictive ability decreases if the dimensionality is too low or too high.

#### Learning rate

The learning rate is a principal factor in ensuring the model’s performance. Too small a learning rate results in excessive convergence time or a failure to converge because the gradient disappeared; too large a learning rate puts the model at the risk of over-approximating the minimum and failing to converge. Therefore, this study used three learning rates, 1e−5, 3e−5, and 5e−5, to determine the sensitivity of this parameter. The experimental results are shown in Table 5.

**Table 5:** Algorithm performance on Movielens-1M dataset under different learning rates.

Learning rates	1e-5	3e-5	5e-5
Pre@20	**0.19052**	0.16436	0.16639
Recall@20	**0.28210**	0.26508	0.26670
NDCG@20	**0.29907**	0.26996	0.27257
HR@20	**0.85630**	0.85397	0.85478

Significant values are in bold.

Table 5 shows that the model performed better when the learning rate was 1e−5. However, when the learning rate was 3e−5 or 5e−5, its performance decreased significantly, and although the training time was significantly reduced, the performance was poor.

#### L2 regularization factor

**Figure 5:**
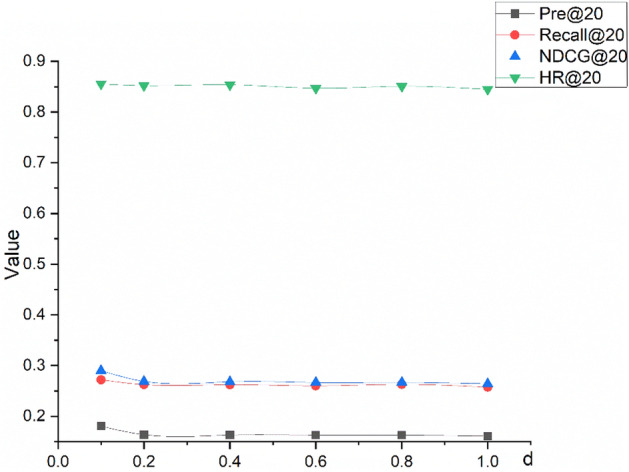
IHDT model performance of Movielens-1M dataset under different regularization coefficients.

Deep learning models usually have superb prediction and fitting capabilities, and thus, they are prone to overfitting. Various regularization techniques can mitigate overfitting to some extent. We used L2 regularization to adjust the overfitting of the model. The experimental results are shown in Fig. 5.

Figure 5 shows that the model achieved a better result when the regularization coefficient was 0.1. The model was robust in response to L2 regularization (i.e., the change in L2 regularization within a specific range did not significantly affect the model’s effectiveness), which also indirectly reflects the stability of the model.

#### Ablation experiments

The proposed model has two improvements over other dual tower recommendation models, based on constructing a heterogeneous recommendation base graph.

1. This paper proposes an interactive dual tower model; that is, when the dual tower model is constructed, the user (item)-related items (users) are injected into the user (item) features for interaction to improve the accuracy.
2. In this paper, we propose to improve the accuracy of the recommendation system through two design improvements. First, we use GNNs with higher-order learning mechanisms on the graph structure data, which shows a powerful feature extraction ability. Second, we converge the information features obtained from the dual tower model as the initial state of the GNN for higher-order fusion to improve the richness of user and item information. To verify the effectiveness and rationality of these two design improvements, we conducted corresponding ablation experiments and compared the difference in performance between the traditional and complete models. The specific completed comparative ablation experiments are as follows.

- ${{\text{IHDT}}}_{noGCN}$: removed the GCN module; it verifies the effect of the model only in the dual tower.
- ${{\text{IHDT}}}_{noEV}$: removed enhancement vector mechanism; it verifies the effect of the model using the traditional dual tower model.

The data used in the ablation experiment were the Movielens-1M dataset. Table 6 shows a comparison of the results of the improved model and the conventional model.

**Table 6:** Validation of ablation experiments on Movielens-1M dataset.

Model	Pre@20	Recall@20	NDCG@20
${ {\text{IHDT}}}_{noGCN}$	0.15972	0.23799	0.24817
${{\text{IHDT}}}_{noEV}$	0.16427	0.26378	0.26967
${\text{IHDT}}$	**0.18052**	**0.27210**	**0.29007**

Significant values are in bold.

Table 6 shows that the two variants of the IHDT model algorithm show different degrees of effect degradation. The interaction-based dual tower model can obtain semantically richer user and item representations. The original dual tower model algorithm cannot obtain the historical interaction behavior of users, thus leading to poor accuracy. In contrast, the higher-order connectivity of GNNs can explore the importance of higher-order neighbors, assign them information on historical interaction behaviors, and enhance user representation and item representation, thus achieving improved accuracy of recommendation results.

#### Results overview

We comprehensively compared the IHDT algorithm with multiple mainstream recommendation algorithms on the public MovieLens data set. Our conclusions are as follows:

1. Accuracy and diversity. IHDT’s precision index (precision) and recall rate (recall) are at the first-tier level among all state-of-the-art models.
2. Response to long-tail demand. Judging from the hit rate indicator, the IHDT algorithm can better discover long-tail items and meet the needs of unpopular preferences. This benefits from the modeling of high-order feature relationships and the mining of implicit associations between users and items, thereby generating personalized recommendation results.
3. Robustness and adaptability. Parameter sensitivity experiments show that IHDT achieves relatively stable performance. The algorithm also performs well across datasets of different sizes. This makes it easy to migrate applications and is very practical.

In summary, the method of combining interactive twin towers with high-order feature representation proposed in this paper shows significant improvement in recommendation performance and application scalability.

### Time cost analysis of introducing GCN high-order learning

The typical twin-tower model is mainly composed of a stack of fully connected layers. Due to the space-for-time strategy, it is very efficient in time. The time complexity of DNN can be expressed as $O(\sum_{i=1}^{k}(U{D}_{i,in}{D}_{i,out}+I{D}_{i,in}{D}_{i,out})$ where U is the number of users, I is the number of items, ${D}_{i,in}{ and D}_{i,out}$ is the in/out representation dimension of layer i, and L is the number of fully connected layers. After introducing interactive learning and GCN, its time complexity increases to $O(L*\left(U{D}^{2}+I{D}^{2}+ED\right))$, where E is the interactive edge Number, D is the representation dimension. Usually, $U{D}^{2}+I{D}^{2}$ and $ED$ are in the same order of magnitude.

## Summary

The paper proposes a new GNN-based recommendation system method, IHDT, that improves the accuracy of the traditional twin-tower recommendation model through interactive twin-tower structure and higher-order feature representation learning. Experimental results show that the IHDT algorithm can significantly improve the recommended accuracy compared with multiple competitive benchmarks.

Despite these advances, there are still several issues that require further study. First, more comprehensive work is needed to compare the computational efficiency of the IHDT method with other methods. Second, this method is currently evaluated only on the movie recommendation dataset; the application of IHDT for other types of recommendations (such as commodities and music) requires further research. Finally, further optimizing the choice of meta-path and user–project feature interaction is also an interesting research direction. Overall, the study provides valuable insights for improving the graph-based recommendation system, but more work needs to be done to promote the development of this field.

## Data Availability

The dataset file of this paper used is available at https://pan.baidu.com/s/1AEh-XNP_nJhxqrKuK0CgeA, with password: a2b4.
